# Contralateral Tension Pneumothorax in One-Lung Ventilation: A Case Report and Systematic Review

**DOI:** 10.7759/cureus.61306

**Published:** 2024-05-29

**Authors:** Angie H Chang, Hongchengcheng Chen, Lei Li, Yirui Hu, Ruoxi Zhang, Xiaopeng Zhang

**Affiliations:** 1 Anesthesiology, Geisinger Medical Center, Danville, USA; 2 Thoracic Surgery, Peking University People's Hospital, Beijing, CHN; 3 Epidemiology and Public Health, Henry Hood Center for Health Research, Geisinger Commonwealth School of Medicine, Danville, USA; 4 Basic Biomedical Sciences, Touro College of Osteopathic Medicine, Middletown, USA

**Keywords:** simulator, lung ultrasound, esophageal stethoscope, contralateral tension pneumothorax, double lumen endobronchial tube, one lung ventilation

## Abstract

Contralateral tension pneumothorax is a rare but fatal complication of one-lung ventilation. The life-saving decompression of pleural space was frequently delayed by the difficult confirmation of diagnosis because of general anesthesia that masks specific clinical presentations when the patient is alert. We reported a case of tension pneumothorax in a patient who underwent thoracic spine instrumentation. There were no contralateral tension pneumothorax cases on file from the search of the Anesthesia Quality Institute Closed Claims Database from 2001 to 2017. We systematically searched PubMed, Ovid MEDLINE, Embase, and Google Scholar. Over the past 30 years, there were 21 single case reports and two case series were retrieved. It was a consensus that difficult confirmation of the diagnosis of contralateral tension pneumothorax is the culprit of delayed life-saving intervention. Difficulty of oxygenation with increasing inspiratory pressure was usually the first sign suggesting contralateral pneumothorax; however, earlier presentations of cardiovascular system failure than respiratory failure have significantly increased the incidence of cardiac arrest and death. It is paramount to maintain a high suspicion of tension pneumothorax. The application of esophageal stethoscope, lung ultrasound, and simulator training may improve the chance of early diagnosis and patient outcome.

## Introduction

One-lung ventilation (OLV) with double-lumen endobronchial tube (DLT) or bronchial blockers has been widely used in thoracic surgery to improve surgical exposures for one-sided thoracotomy or video-assisted thoracoscopic procedures. In addition, OLV was applied more and more in complex thoracic spine procedures, aortic aneurysm repair, and minimally invasive cardiac surgeries as well. The management of airway instrumentation, general anesthesia, and dependent-lung ventilation could be challenging, and the diagnoses of conditions such as rare and life-threatening contralateral tension pneumothorax may be delayed due to the patient’s non-specific presentations, including hypoxemia, hypotension, reduced airway compliance, insufficient deflation of the lung on surgical side. We reported a case of contralateral tension pneumothorax in a patient who underwent thoracic spine instrumentation with one-lung ventilation. Our search of the Anesthesia Quality Institute Closed Claims Database from 2001 to 2017 did not reveal any similar cases.

In addition, Ovid MEDLINE, PubMed Central, Embase, and Google Scholar were searched from 1990 until April 2024 for clinical trials, prospective and retrospective cohort studies, case reports, case series related to clinical presentations, etiologies, diagnoses and therapeutic management, and outcomes of dependent side tension pneumothorax in patients under procedures facilitated by OLV.

## Case presentation

A 53-year-old female, with a past medical history of thoracolumbar spine stenosis and type 2 diabetes mellitus (DM), a height of 161.3 cm, weight of 109 kg, body mass index (BMI) 38.72 kg/m^2^, history of gastric bypass, well-controlled asthma, and history of difficult intubation, was scheduled for open left thoracotomy for T9, T10 anterior thoracic spine discectomy, and spinal fusion under neurophysiological monitoring. Her preoperative stress echocardiography was positive for a small area of apical ischemia. Her preoperative vital signs are blood pressure (BP) 137/70 mmHg, heart rate (HR) 68/min, and oxygen saturation (SpO_2_) 98%. General anesthesia was induced with propofol 200 mg, fentanyl 50 µg, succinylcholine 140 mg, and lung compliance was normal upon mask ventilation. The anesthesiologist failed to insert a size 37 French gauge (Fr) left-side double-lumen endobronchial tube (DLT), after several unsuccessful attempts, a 35 Fr left side DLT was successfully placed through exchanging of a 7.5 mm single-lumen endotracheal tube with a cook airway exchanger. The position of DLT was confirmed by fiberoptic bronchoscopy. A right radial arterial line was placed, through the case, and total intravenous anesthesia (TIVA) was maintained by ketamine, propofol, and phenylephrine infusion without muscle paralytics, to accommodate the neurophysiological monitoring.

The patient was turned to right lateral decubitus position, after confirming the DLT position by fiberoptic bronchoscopy, volume-controlled one-lung ventilation with tidal volume of 575 mL was initiated by clamping the bronchial side. The peak airway pressure was increasing slowly, about one hour after skin incision, the anesthesiologist noticed that the high airway pressure alarm went off, the peak inspiratory pressure (PIP) was 35 cmH_2_O, the delivered tidal volume was only 300 mL, the respiratory rate increased from 12/min to 18/min, positive end-expiratory pressure (PEEP) was set and unchanged at 6 cmH_2_O. The SpO_2_ slowly decreased to 88% despite increased inspiratory oxygenation to 100%, low dependent lung compliance was felt by hand ventilation. It was difficult to auscultate the respiratory sound of right lung using surface stethoscope, there was a questionable wheezing sound. Repeated bronchoscopy confirmed correct DLT position, although the left bronchus was found edematous. After 5 min, it was unable to ventilate the right lung with increased PIP to above 60 cmH_2_O and SpO_2_ was exponentially reduced to less than 80%. The lung isolation was suspended, through hand ventilation with 100% oxygen, and SpO_2_ quickly recovered. The PIP decreased to 27~30 cmH_2_O with delivered TV ~500 mL. A 0.5 mL 2.25% racemic epinephrine was given through the DLT, and the patient’s BP increased to 218/120 mmHg, requiring treatment of two bolus injections of 400 µg nitroprusside. There was no improvement in respiratory symptoms. After discussing with the surgeon, because of suspicion of pneumothorax, the decision was made to abort one-lung ventilation, close the wound, and confirm the diagnosis with a chest x-ray. The ventilation was switched to pressure control mode with an inspiratory pressure setting at 14 cmH_2_O. However, despite this setting, the tidal volume could barely reach 400 mL. The arterial blood gas indicated mild respiratory acidosis with a pH of 7.309 units, pCO_2_ of 50.5 mmHg, pO_2_ of 228 mmHg, and arterial oxygen content of 14.5%. She required an increasing dosage of phenylephrine to support her blood pressure.

**Figure 1:**
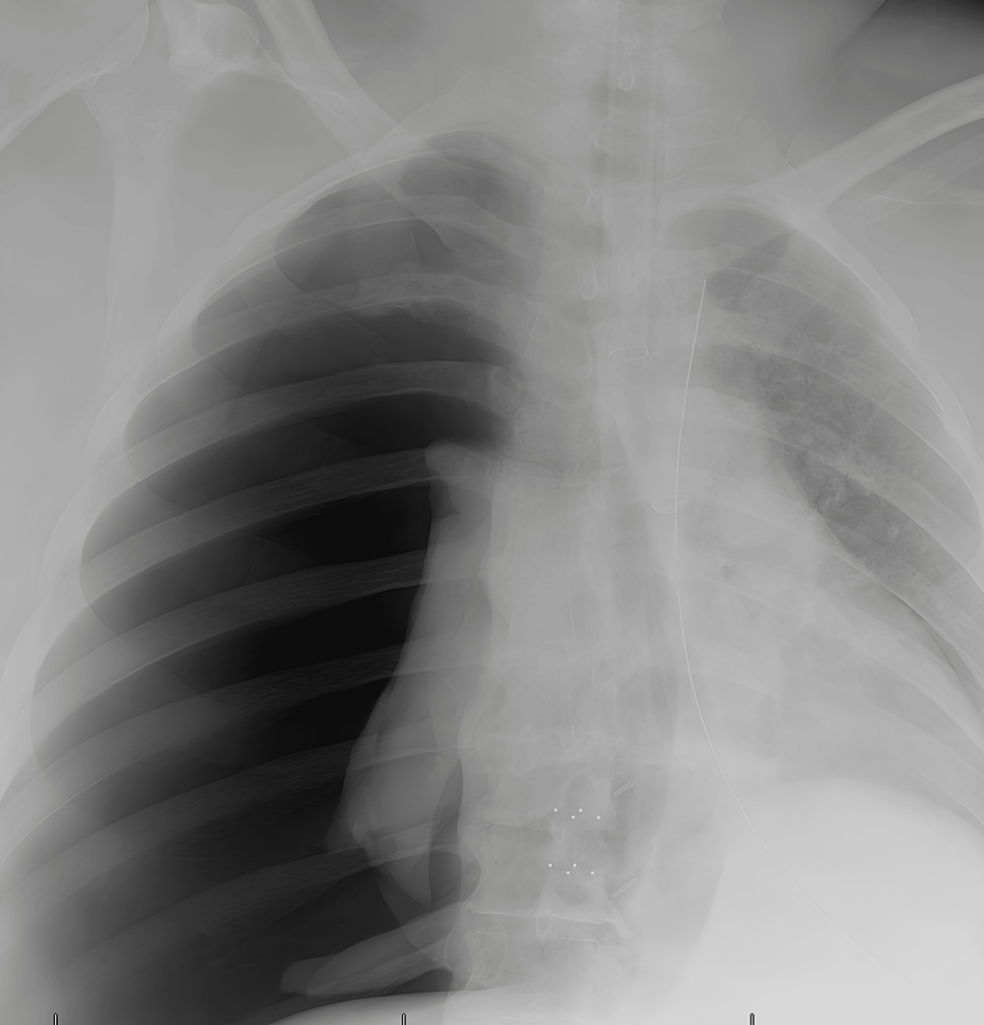
Right lung was completely compressed on bedside chest x-ray.

After skin closure patient was quickly turned to supine position, there was no breathing sound on the right side. The room chest x-ray showed a completely collapsed right lung, diagnosis of tension pneumothorax with a significant left shift of mediastinum was confirmed (Figure 1), an emergent chest tube was placed in the right fifth intercostal space, the patient’s hemodynamic status immediately improved, airway pressure was normalized, respiratory sound equalized on both side, and phenylephrine was no longer required for supporting BP. Suspicious of airway injury, the patient was observed postoperatively in the surgical ICU. She was extubated on postoperative day one after confirming negative for an air leak. The rest of the hospitalization was uneventful.

## Discussion

Inquiry of Anesthesia Quality Institute Closed Claims Database

In collaboration with the American Society of Anesthesiologists, we searched the Closed Claims Database of the Anesthesia Quality Institute. Between 2001 and 2017, there was no claim filed for contralateral pneumothorax related to one-lung ventilation, although there were 22 cases of pneumothorax after single-lumen endotracheal intubation and mechanical ventilation, of these, two cases were confirmed to develop one-sided tension pneumothorax. One patient, scheduled for laparoscopic Nissen fundoplication, had intraoperative right pneumothorax from the injury of placement of an esophageal dilator, the patient survived after stormy postoperative hospital cause, and this case ended up settling with high figures. The other patient, scheduled for thyroid lobectomy, developed right-side tension pneumothorax possibly from tracheal intubation, mechanical ventilation, or injury from pericardiocentesis, patient died of hypoxemic encephalopathy after cardiac arrest.

Tension pneumothorax is a life-threatening condition that requires emergent decompressive intervention based on prompt recognition of the clinical signs of anxiety and deteriorating mental status, tachypnea, difficult breathing, distant breathing sound, hyperresonance from percussion, and hemodynamic instability when the patient is alert. However, the onset and progress of tension pneumothorax under general anesthesia, due to the multiple confounding issues of challenging endotracheal or endobronchial intubation, surgical positioning, and positive pressure mechanical ventilation, is usually insidious and non-specific, making the confirmation of diagnosis more difficult, resulting delayed life-saving decompression of pleural space to prevent cardiac arrest with high morbidity and mortality. 

Contralateral tension pneumothorax is quite rare in patients who underwent a procedure with one-lung ventilation. Data from recent case series suggest the incidence was 0.16% in the general population [1]. In patients with intrinsic lung diseases, e.g., severe emphysema, the incidence could be as high as 4.2% [2]. We systematically searched the major indexed databases from 1990 until today, there are 21 published case reports and two case series of contralateral tension pneumothorax from one-lung ventilation (Table 1).

**Table 1:** Summary of characteristics of published cases of contralateral tension pneumothorax. TP: tension pneumothorax; AE: airway exchanger; BP: blood pressure; OLV: one-lung ventilation; BS: breathing sound; SaO_2_: oxygen saturation; EtCO_2_: end-tidal CO_2_; PIP: peak inspiration pressure; PEA: pulseless electric activity; HR: heart rate; CXR: chest x-ray; DLT: double-lumen endobronchial tube; CPR: cardiopulmonary resuscitation; BB: bronchial blocker

Author	Year	Age	AE	First sign of TP	Hemodynamic change	Diagnostic methods	Cardiac arrest	Outcome	Mechanism of TP
Gabbott and Carter [3]	1990	74 years	No	↓ SaO_2_	Negative	CXR	Negative	Survived	Barotrauma
Laishley and Aps [4]	1991	63 years	No	↓ SaO_2_	Negative	Clinical diagnosis	Negative	Survived	Mispositioned DLT
Handel and Jellicoe [5]	1993	72 years	Yes	↑ PIP	↓ BP	Clinical diagnosis	Negative	Survived	Inconclusive
Stühmeier et al. [6]	1997	63 years	No	↓ SaO_2_	Negative	CXR	Negative	Survived	Inconclusive
Zollinger et al. [2]	1997	Unknown	No	↓ SaO_2_	↓ BP	Clinical diagnosis	Negative	Survived	Barotrauma
Sivalingam and Tio [7]	1999	15 years	No	↓ SaO_2_	↓ HR, ↓BP, ↓ET CO_2_	CXR	Negative	Survived	Mispositioned and sized DLT
Roush et al. [8]	2000	13 years	No	↑ HR, ↓SaO_2_	VA-ECMO	Fluoroscope	Negative	Survived	Injury from surgery
Fossard et al. [9]	2001	28 years	No	↓ SaO_2_	↓ HR, ↓BP, ↓ET CO_2_	Clinical diagnosis	Negative	Survived	Barotrauma
Malik et al. [10]	2002	76 years	No	↑ PIP	Negative	CXR	Negative	Survived	Mispositioned and sized DLT
Weng et al. [11]	2002	76 years	No	↓ SaO_2_	PEA	Clinical diagnosis	Yes, CPR	Survived	Mispositioned and sized DLT
Huang et al. [12]	2005	34 years	No	↑ PIP	PEA, V-tach	Clinical diagnosis	Yes, CPR	Deceased	Wrong sized DLT
Kim et al. [13]	2005	59 years	No	↓ Tidal volume	↓ BP	CXR	Negative	Survived	Unspecified
Finlayson and Brodsky [14]	2008	57 years	Yes	↑ PIP	Negative	CXR	Negative	Survived	Barotrauma
Akindipe et al. [15]	2008	55 years	No	↓ BP, ↑ EtCO_2_	PEA	Postmortem CXR	Yes, CPR	Deceased	Barotrauma
Akindipe et al. [15]	2008	53 years	No	↓ BP	PEA	CXR	Negative	Deceased	Barotrauma
Akindipe et al. [15]	2008	59 years	No	Tachycardia	↑ HR, ↓ BP	CXR	Negative	Survived	Unspecified
No et al. [16]	2012	79 years	No	↑ PIP	↓ BP	CXR	Negative	Survived	BB injury
Kenta et al. [17]	2015	63 years	Yes	↓ SaO_2_, ↑PIP	PEA	Clinical diagnosis	Yes CPR	Survived	AE
Agrawal and Nambala [18]	2016	70 years	No	RV compression	↓ BP	Clinical diagnosis	Negative	Survived	Surgery
Arai et al. [19]	2018	72 years	No	↓ SaO_2_	↓ BP	Clinical diagnosis	Negative	Survived	Frequent suctioning
Huang et al. [20]	2018	68 years	No	↓ SaO_2_	↓ BP, ↓ HR	CXR	Negative	Survived	Barotrauma
Hoechter et al. [1]	2018	26 years	No	Cardiac arrest	PEA	Clinical diagnosis	Yes, CPR	Deceased	Barotrauma
Hoechter et al. [1]	2018	49 years	No	↓ SaO_2_	Negative	Clinical diagnosis	Negative	Survived	Unspecified
Hoechter et al. [1]	2018	65 years	No	↓ SaO_2_	↓ BP	Chest CT	Negative	Deceased	Unspecified
Hoechter et al. [1]	2018	57 years	No	↓ BP	VA-ECMO	CXR	Negative	Deceased	Unspecified
Lee et al. [21]	2020	75 years	No	↓ SaO_2_, ↑PIP	PEA	CXR	Yes, VA-ECMO	Survived	Barotrauma
Tani et al. [22]	2021	57 years	No	↓ SaO_2_	↑ HR, ↓ BP	CXR	Negative	Survived	Entry of CO_2_
Zhang et al. [23]	2022	36 weeks	Yes	↑PaCO_2_	PEA	Clinical diagnosis	yes, CPR	Survived	Barotrauma
Baek et al. [24]	2023	84 years	Yes	↓ SaO_2_	↓ BP	CXR	Yes, VA-ECMO	Deceased	Mispositioned and sized DLT
Current case	2023	53 yes	Yes	↓ SaO_2_, ↑PIP	↓ BP	CXR	Negative	Survived	AE, Barotrauma

Among the 29 cases listed, including the present case, one-lung ventilation was established with a double-lumen endobronchial tube (DLT, 82.8%), bronchial blocker (6.9%), or mainstem bronchus intubation (3.4%). Twenty-six had contralateral pneumothorax (89.7%) and three progressed to bilateral pleural space involvement (10.3%). The mechanisms of contralateral tension pneumothorax are injuries from airway exchanger (31%), frequent suction, pleural damage from surgery, and jet ventilation; barotrauma from high inspiratory pressure (27.6%) and jet ventilation (3.4%); as well as mispositioned or wrong-sized double-lumen tube (17.3%). Eight patients developed cardiac arrest (27.6%) seven died and the calculated mortality was 24.1%. Notably, the mortality was significantly related to cardiovascular failure, not respiratory symptoms, as the earliest presentation (p=0.018). There was no significant impact of mortality from ventilation mode, peak inspiratory pressure (PIP), positive end-expiratory pressure (PEEP), and application of inotrope. Confirmation of the diagnosis and the timely decompression are most important for reducing morbidity and mortality.

Tension pneumothorax frequently has clinical presentations of early respiratory symptoms and late signs of cardiovascular failure. There are 25 (86.2%) patients who presented initially with decreased oxygen saturation, decreased tidal volume, increased PIP, and failed one-lung ventilation; however, these symptoms are quite non-specific, they could be caused by trachea-bronchospasm, pulmonary embolism, aspiration, pulmonary edema, and severe anaphylactic reaction. In addition, the surgical drape and lateral decubital position make the surface auscultation of dependent lung sound and chest percussion unreliable for confirming diagnosis. The findings of fiberoptic bronchoscopy, including the collapse of bronchus, increased bronchus secretion, and the abovementioned non-specific respiratory presentations could make the early clinical differential diagnosis more difficult. In this series, there were only nine (31%) cases that had confirmed pneumothorax by clinical diagnosis, the other cases were confirmed at the end of the surgery by chest x-ray, fluoroscopy, or chest CT. Since 70% of the patients were at risk of fatal delayed decompression of pleural space resulting in cardiovascular system failure, reliable tests with high sensitivity and specificity are critical for reducing morbidity and mortality.

Dean et al., in their case report of bilateral tension pneumothorax from mechanical failure of the anesthesia machine, emphasized the significance of the application of an esophageal stethoscope, which facilitated the auscultation of fainting of heart sound along with distant respiratory sound indicating pneumothorax. The chest tube was placed according to clinical diagnosis without delayed confirmation with images since the confirmation of the chest x-ray might delay the life-saving pleural decompression [25]. Although it was not studied by randomized clinical trial, esophageal stethoscope would be quite valuable for monitoring intraoperative pneumothorax because it is very close to the dependent lung.

Recent randomized clinical trials have shown that the application of thoracic ultrasound has a sensitivity of 90.0%, specificity of 98.9%, and diagnostic accuracy of 96.7% for the diagnosis of pneumothorax [26]. Studies also suggest replacing fiberoptic bronchoscopy with thoracic ultrasound for confirmation of proper lung isolation based on the demonstration of the image of correct lung exclusion [27,28]. With proper training, we believe that thoracic ultrasound is extremely helpful for confirming diagnosis when pneumothorax is suspected. Because of the increasing application of lung isolation in minimally invasive cardiac surgery, the use of transesophageal echocardiogram (TEE) may be potentially diagnostic in patients with contralateral tension pneumothorax. Agrawal and Nambala reported TEE images of a mass effect on the right atrium within a suspicious confined space and the signs of cardiac tamponade. It is hard to draw conclusions on the direct diagnostic value of TEE on tension pneumothorax based on a single case report; however, for a patient with one-lung ventilation, TEE could provide evidence of significant hemodynamic instability suggesting impending cardiac arrest and tension pneumothorax must be ruled out first.

The decompression of pleural space as early as possible is the only way to prevent cardiac arrest and death. The key is for clinicians to maintain high vigilance for tension pneumothorax. However, due to the rarity of this deadly complication, clinicians tend to deny or downgrade it on their list of differential diagnoses. A study of anesthesia simulation by Gaba points out that this is typically a fixation error that occurs frequently due to cognitive failure in facing the clinical evidence that is contradictory to the initial assessment [29]. The incidence of fixation error in response to crucial events is 63%; therefore, this may play an important role in the inappropriate management of tension pneumothorax. Handel and Jellicoe pointed out that acknowledgment of fixation error illustrates the need for careful evaluation of the available clinical evidence when the clinical problem cannot be resolved by initial intervention, like the endotracheal application of racemic epinephrine [5]. Given the fact that only 28 cases were published over three decades globally, attending the anesthesia simulator training may be the best way to reduce the incidence of delayed intervention of tension pneumothorax.

## Conclusions

In conclusion, contralateral pneumothorax during OLV is a deadly complication, failure of early confirmation of diagnosis due to non-specific presentations is responsible for the high morbidity and mortality. The difficulty of oxygenation with increasing PIP was usually presented earlier than hemodynamic instability; however, earlier presentations of cardiovascular system failure than respiratory failure had significantly increased the incidence of cardiac arrest and death. It is paramount for physicians to maintain high suspicion of tension pneumothorax. The application of esophageal stethoscope, lung ultrasound, and simulator training may help to improve the chance of early diagnosis and patient outcome.
